# PM_10_ and *Pseudomonas aeruginosa*: effects on corneal epithelium

**DOI:** 10.3389/fcimb.2023.1240903

**Published:** 2023-10-05

**Authors:** Mallika Somayajulu, Sharon A. McClellan, Farooq Muhammed, Robert Wright, Linda D. Hazlett

**Affiliations:** Department of Ophthalmology, Visual and Anatomical Sciences, Wayne State University, School of Medicine, Detroit, MI, United States

**Keywords:** PM10, *P. aeruginosa*, cornea, epithelium, human

## Abstract

**Purpose:**

*In vivo* data indicate that mouse corneas exposed to PM_10_ showed early perforation and thinning after infection with *Pseudomonas aeruginosa*. To understand the mechanisms underlying this finding, we tested the effects of PM_10_ and the mitochondria targeted anti-oxidant SKQ1 in immortalized human corneal epithelial cells (HCET) that were challenged with *Pseudomonas aeruginosa* strain 19660.

**Methods:**

Mouse corneas were infected with strain 19660 after a 2 week whole-body exposure to PM_10_ or control air and assessed by clinical scores, slit lamp photography and western blot. HCET were exposed to 100μg/ml PM_10_ for 24h before challenge with strain 19660 (MOI 20). A subset of cells were pre-treated with 50nM SKQ1 for 1h before PM_10_ exposure. Phase contrast microscopy was used to study cell morphology, cell viability was measured by an MTT assay, and ROS by DCFH-DA. Levels of pro-inflammatory markers and anti-oxidant enzymes were evaluated by RT-PCR, western blot and ELISA. Reduced glutathione (GSH) and malondialdehyde (MDA) levels were evaluated by assay kits.

**Results:**

*In vivo*, whole body exposure to PM_10_ vs. control air exposed mouse corneas showed early perforation and/or corneal thinning at 3 days post infection, accompanied by increased TNF-α and decreased SOD2 protein levels. *In vitro*, PM_10_ induced a dose dependent reduction in cell viability of HCET and significantly increased mRNA levels of pro-inflammatory molecules compared to control. Exposure to PM_10_ before bacterial challenge further amplified the reduction in cell viability and GSH levels. Furthermore, PM_10_ exposure also exacerbated the increase in MDA and ROS levels and phase contrast microscopy revealed more rounded cells after strain 19660 challenge. PM_10_ exposure also further increased the mRNA and protein levels of pro-inflammatory molecules, while anti-inflammatory IL-10 was decreased. SKQ1 reversed the rounded cell morphology observed by phase contrast microscopy, increased levels of MDA, ROS and pro-inflammatory molecules, and restored IL-10.

**Conclusions:**

PM_10_ induces decreased cell viability, oxidative stress and inflammation in HCET and has an additive effect upon bacterial challenge. SKQ1 protects against oxidative stress and inflammation induced by PM_10_ after bacterial challenge by reversing these effects. The findings provide insight into mechanisms underlying early perforation and thinning observed in infected corneas of PM_10_ exposed mice.

## Introduction

Airborne particulate matter (PM) with an aerodynamic diameter <10 microns (PM_10_) is a complex mixture of solids and aerosols and a major component of air pollution (Crinnion, 2017). Exposure to PM_10_ has been linked to adverse health effects (Pope et al., 2002) and increased mortality (Pope and Dockery, 2006), chronic cardiopulmonary diseases (Nurkiewicz et al., 2006; Liu et al., 2009; Miller et al., 2009; Zhang et al., 2021), chronic obstructive pulmonary disease (Churg et al., 2003), diabetes (Wang et al., 2014) and cancer (Brunekreef and Holgate, 2002). However, few studies have investigated its effects on the eye, although the ocular surface is continuously exposed to the pollutants (Hao et al., 2022). Recently, epidemiological studies have linked PM_10_ exposure to an increased rate of outpatient visits for eye discomfort (Chang et al., 2012), elevated risk of Sjogren’s syndrome (Zhang et al., 2022) and worsened tear film stability in patients with dry eye disease (Kim et al., 2020).

PM can disrupt the corneal epithelial barrier function (Ko et al., 2021), thus rendering the ocular surface more prone to infections. The effects of PM_10_ on ocular infections have not been well investigated and there is little information on how PM_10_ affects disease progression and severity of ocular infections. A study from Argentina demonstrated that urban particulate matter exacerbated inflammation in a mouse model of acute herpes simplex keratitis (Sendra et al., 2021). An epidemiological study from South Korea, showed that exposure to high concentration of PM_10_ correlated with increased outpatient visits for ocular diseases, including emergency room visits for keratitis (Lee et al., 2018). And it is well known that dry eye increases the propensity for microbial infection (Klopfer, 1989; Torricelli et al., 2014; Mo et al., 2019; Manisalidis et al., 2020).

Microbial keratitis is a vision threatening disorder and is related to several risk factors, which include extended use of soft contact lenses (Stapleton and Carnt, 2012; Hazlett et al., 2021), ocular surface disease (O'Neill et al., 2014), ocular surgery (Keay et al., 2006) and immunosuppression (Otri et al., 2013). Pathogens such as *Pseudomonas aeruginosa* (*P. aeruginosa*) are often the causative agent in eye infections (Hilliam et al., 2020). *P. aeruginosa* is a gram negative bacterium and a leading cause of contact lens–induced microbial keratitis (Green et al., 2008). If untreated, these infections can lead to ocular pain, stromal destruction, corneal thinning, and/or perforation, eventually leading to vision loss (Ekanayaka et al., 2016). A critical antimicrobial mechanism employed by host immune cells to combat microbial infection is oxidative burst, a process which involves production of reactive oxygen (ROS) and reactive nitrogen (RNS) species (Lambeth, 2004). However, excessive production of ROS/RNS by activated immune cells generates a cytotoxic environment that contributes to inflammatory responses which are noxious to organs (Novaes et al., 2019). PM_10_ has been shown to exert its toxic effects by inducing free radical production and inflammation in cornea (Yoon et al., 2018; Somayajulu et al., 2023). Mitochondria are the main sources of free radical generation (Kowalczyk et al., 2021) and we have recently shown that PM_10_ exposure causes mitochondrial dysfunction by elevating superoxide free radicals and decreasing ATP levels (Somayajulu et al., 2023). Studies are ongoing in search of potential therapeutics to protect against oxidative and inflammatory damage induced by PM_10_. One such therapeutic compound is SKQ1 (10-(6′-plastoquinonyl) decyltriphenylphosphonium), a mitochondria-specific anti-oxidant that accumulates in the inner mitochondrial membrane (Skulachev, 2013) and has proven effective against oxidative stress in animal models of ischemia/reperfusion (Kezic et al., 2016), aging (Skulachev, 2011) and neurodegeneration (Genrikhs et al., 2015). It has been formulated as an eye drop (Visomitin) to prevent anesthesia-induced dry eye syndrome in patients after long-term general anesthesia or ocular surgery (Zernii et al., 2017). Recently, the use of Visomitin for the treatment of dry eye disease (Brzheskiy et al., 2015) has been tested in a phase 3 clinical trial in the USA (Ousler et al., 2022) with good outcome.

The purpose of this study is to determine whether exposure of the eye to PM_10_ has an adverse effect on the development and progression of *P. aeruginosa* induced keratitis. Because we have observed that *P. aeruginosa* infection of the mouse cornea after PM_10_ exposure caused earlier corneal perforation and/or thinning, we sought to determine the mechanism underlying this observation *in vitro* using immortalized human corneal epithelial cells. We also used this *in vitro* system to test the beneficial effects of SKQ1.

## Materials and methods

### Mice

Female C57BL/6 mice, 8 weeks of age, were purchased from the Jackson Laboratory (Bar Harbor, ME) and housed in accordance with the National Institutes of Health guidelines. Mice were humanely treated and in compliance with both the ARVO Statement for the Use of Animals in Ophthalmic and Vision Research and the Institutional Animal Care and Use Committee of Wayne State University (IACUC-21-09-4042).

### Whole body exposure to PM_10_

Experiments in this study were performed with PM_10_ purchased from the National Institute of Standards and Technology, (NIST) (Standard Reference Material (SRM) 2787). A whole-body exposure chamber (CH Technologies, Westwood, NJ) was used for this study as previously described (Somayajulu et al., 2023). Briefly, mice were exposed to control air or an acute, high dose of PM_10_ (500µg/m^3^) for 2 weeks for 3h/day/5days/week and rested on the weekends. The dosage used in this study was based upon mean PM_10_ concentrations measured in the winter, ranging as high as 494µg/m3 in 5 Chinese cities (Cao et al., 1988).

### Bacterial culture and infection

As previously described, *P. aeruginosa* strain ATCC (American Type Culture Collection [ATCC] Manassas, VA, USA) 19660, a cytotoxic strain (Ekanayaka et al., 2016) was grown in peptone tryptic soy broth (PTSB) medium in a rotary shaker water bath at 37°C and 150 rpm for 18h to an optical density (measured at 540 nm) between 1.3 and 1.8. Bacterial cultures were centrifuged at 5,500g for 10 min. Bacterial pellets were washed once with sterile saline, recentrifuged, resuspended, and diluted in sterile saline. Mice exposed to PM_10_ and control air were infected with strain 19660 24h after the last chamber exposure. Mice were infected as described previously (Moon et al., 1988; Ekanayaka et al., 2018). Briefly, mice were anesthetized with ether and placed under a stereoscopic microscope at 40 × magnification. The left cornea was scarified, and 5μl containing 1 × 10^6^ colony-forming units (CFU)/μl of the bacterial suspension was applied topically.

### Ocular response to bacterial infection

As previously described, clinical scores were designated as follows: 0 = clear or slight opacity, partially or fully covering the pupil; +1 = slight opacity, fully covering the anterior segment; +2 = dense opacity, partially or fully covering the pupil; +3 = dense opacity, covering the entire anterior segment; and +4 = corneal perforation or phthisis (Moon et al., 1988). Each mouse was scored in masked fashion at 1, and 3 days post infection (p.i.) for statistical comparison and photographed (3 days p.i.) with a slit lamp to demonstrate disease.

### Tissue culture and treatments

HCET cells (HCE-2 [50.B1], ATCC, Gaithersburg, MA) were cultured in Keratinocyte-serum free (KSF) medium (Gibco, Grand Island, NY) with 5ng/ml human recombinant EGF, 0.05mg/ml bovine pituitary extract, 0.005mg/ml insulin, and 500ng/ml hydrocortisone as previously described (Somayajulu et al., 2023). Cells were treated with PM_10_ (0, 25, 50, 100, 200, 500, 800 and 1200µg/ml for 24h for the MTT assay. For all other experiments, cells were incubated with 100μg/ml (per cell viability data from dose curve; 75-80% viability) PM_10_ at 37°C and 5% CO_2_ for 24hr. To investigate the combined effects of PM_10_ on *P. aeruginosa* infected cells, a subset of cells were challenged with strain 19660 at a multiplicity of infection (MOI) of 20 for 3h. To assess the effects of SKQ1 (BOC Sciences, Shirley, NY, USA), another subset of cells were incubated with 50nM SKQ1, 1h before PM_10_ exposure (Somayajulu et al., 2023) and then challenged with strain19660 at similar MOI. Another group of HCET were challenged with strain 19660 at a MOI of 20 for 3h to assess the effects of bacteria alone on these cells. Phase contrast microscopy was used to photograph cell preparations using a Leica EL 6000 microscope (Deerfield, IL, USA). All the images were acquired at the same magnification and processed similarly.

### Cell viability assays

An MTT 3-(4, 5-dimethylthiazol-2-yl)-2,5-diphenyltetrazolium bromide (ThermoFisher Scientific, Grand Island, NY) assay was used to test the effects of PM_10_ on HCET viability as reported before (Somayajulu et al., 2023). Briefly, 15,000 HCET cells were seeded in a 96 well plate, treated with 0, 25, 50, 100, 200, 500, 800 and 1200µg/mL PM_10_ for 24h or PM_10_ (100µg/ml) ± 50nM SKQ1 ± strain 19660 as described above in “Tissue culture and treatments”. At the end of the treatment, 5mg/ml MTT reagent was added to each well and incubated at 37°C for 4h and media removed. Dimethyl sulfoxide (DMSO, 100%) was added (50µl/well) and optical density was read at 540nm using a SpectraMax M5 microplate reader (Molecular Devices, Sunnyvale, CA). Data are shown as % cell viability + SD. To confirm the MTT data, a trypan blue exclusion cell viability assay was also performed using Countess cell counting chamber slides (Invitrogen, Carlsbad, CA) per the manufacturer’s protocol. Briefly, cells were treated as mentioned in “Tissue culture and treatments” and trypsinized 24h post treatment, centrifuged, resuspended in KSF media and incubated 1:1 with trypan blue. A 10μl aliquot was placed on the chamber slide and viable cells were counted using a Countess automated cell counter (Invitrogen, Carlsbad, CA). Data are shown as % cell viability + SD.

### Western blot analysis

HCET were treated with 100µg/ml PM_10_ ± strain 19660 ± SKQ1, washed with ice-cold 0.1M PBS (pH 7.4), lysed in RIPA buffer with protease and phosphatase inhibitors (SantaCruz Biotech, Dallas, TX), incubated on ice 20min, centrifuged at 12,000g at 4°C for 10min and supernatant collected. Total protein was determined from the supernatants using a BCA protein kit (ThermoFisher Scientific). Briefly, samples (20μg) were run on SDS-PAGE in Tris-glycine-SDS buffer and electro-blotted onto nitrocellulose membranes (BioRad). After blocking for 1h in 5% MTBST (Tris Buffer saline containing 0.05% Tween 20 (TBST) and 5% nonfat milk), membranes were probed with primary antibodies: rabbit anti-mouse pNFκB, NFκB, iNOS, COX2 and TNF-α (1:1000; Cell Signaling Technology, Danvers, MA), in 2% MTBST overnight at 4°C. The membranes were then washed three times with TBST and incubated with HRP-conjugated anti-rabbit secondary antibody (1:2000; Cell Signaling Technology) diluted in 5% MTBST at room temperature for 2h. Bands were developed with Supersignal West Femto Chemiluminescent Substrate (ThermoFisher Scientific), visualized using an iBright™ CL1500 Imaging System (ThermoFisher Scientific), and normalized to β-tubulin (1:1000; Abcam, Waltham, MA) and intensity quantified using ImageJ software. Data are shown as mean integrated density values (IDV) + SD.

### RT-PCR

Total RNA was isolated from cells treated with 100µg/ml PM_10_, ± strain 19660 ± SKQ1 and control HCET (n=3/group/treatment) using RNA STAT-60 (Tel-Test, Friendswood, TX) per the manufacturer’s instructions as reported before (Huang et al., 2005). Briefly, reverse transcription was performed with one μg of RNA from each sample using Moloney-murine leukemia virus (M-MLV) reverse transcriptase (Invitrogen, Carlsbad, CA) to produce a cDNA template. A 2μl aliquot of diluted cDNA (1:20 in DEPC-treated water) was used for the RT-PCR reaction. SYBR green/fluorescein PCR master mix (Bio-Rad Laboratories, Richmond, CA) and primer concentrations of 10μM were used in a total 10μl volume. After a pre-programmed hot start cycle (3min at 95°C), PCR amplification was repeated for 45 cycles with parameters: 15s at 95°C and 60s at 60°C. Levels of high mobility group box 1 (HMGB1), inducible nitric oxide synthase (iNOS), cyclooxygenase 2 (COX2), interleukin (IL)-1β, IL-6, IL-10, toll-like receptor (TLR)4, tumor necrosis factor (TNF)-α, nicotinamide adenine dinucleotide phosphate (NADPH) quinone dehydrogenase 1 (NQO1), glutathione peroxidase (GPX4), glutamate-cysteine ligase modifier subunit (GCLM), and catalase were tested by real-time RT-PCR (CFX Connect real-time PCR detection system; Bio-Rad Laboratories). The fold differences in gene expression were calculated using the formula: Fold change= 2^-ΔΔCT^ and expressed as relative mRNA levels + SD. Primer pair sequences used are shown in **Table 1**.

**Table 1:** Nucleotide sequence of the specific primers used for PCR amplification (Human).

Gene	Nucleotide Sequence	Primer	GenBank
*18s rRNA*	5’- CGG CTA CCA CAT CCA AGG AA -3’ 5’- GCT GGA ATT ACC GCG GCT-3’	F R	NR_003286.4
*HMGB1*	5’- TGG CCA AGG AAT CCA GCA GTT -3’ 5’- CTC CTC CCG ACA AGT TTG CAC -3’	F R	NM_001313893.1
*IL-1β*	5’- CCT GTC CTG CGT GTT GAA AGA-3’ 5’- GGG AAC TGG GCA GAC TCA AA-3’	F R	NM_000576.3
*IL-6*	5’- GTA GCC GCC CCA CAC AGA CAG CC-3’ 5’- GCC ATC TTT GGA AGG TTC-3’	F R	NM_000600.5
*IL-10*	5’- GCT GGA GGA CTT TAA GGG TTA CCT3’ 5’- CTT GAT GTC TGG GTC TTG GTT CT-3’	F R	NM_000572.3
*TNF-α*	5’- CCC CAG GGA CCT CTC TCT AAT C-3’ 5’- GGT TTG CTA CAA CAT GGG CTA CA-3’	F R	NM_000594.4
*TLR4*	5’- CAG AAC TGC AGG TGC TGG-3’ 5’- GTT CTC TAG AGA TGC TAG-3’	F R	NM_138554.3
*COX2*	5’- TTC AAA TGA GAT TGT GGG AAA ATT GCT-3’ 5’- AGA TCA TCT CTG CCT GAG TAT CTT-3’	F R	NM_000963.4
*iNOS*	5’- GGT GGA AGC GGT AAC AAA GG-3’ 5’- TGC TTG GTG GCG AAG ATG A-3’	F R	NM_000625.4
*NQO1*	5’- GGG CTC AAG AGG CCA CTT AG-3’ 5’- ACC AAA CAA GTT AAG TCC CT-3’	F R	NM_000903.3
*GPX4*	5’- CCT TCC CGT GTA ACC AGT TC -3’ 5’- ACT TGG TGA AGT TCC ACT TGA TG-3’	F R	NM_001039847.3
*GCLM*	5’- TGG CCT AGG TAT CAG GGT AAT G-3’ 5’- AGT AAA TCC CAG CTA CTC CAG TT-3’	F R	NM_001308253.2
*CATALASE*	5’- TGG TAA ACT GGT CTT AAA CCG GAA TC -3’ 5’- GGC GGT GAG TGT CAG GAT AGG-3’	F R	NM_001752.4

F, forward; R, reverse.

### ROS assay

Cells (n=3/group/treatment) were treated as described in tissue culture and lysed in RIPA buffer with protease and phosphatase inhibitors (SantaCruz Biotech, Dallas, TX). A previously published method was used to detect ROS (Carion et al., 2018). Briefly, 8μl of cell lysates were incubated in a reaction buffer [130mM KCl, 5mM MgCl_2_, 20mM NaH_2_PO_4_, 20mM Tris-HCl, pH 7.4, 30mM glucose, 7.5uM 2’,7’-dichlorofluorescein diacetate (DCFH-DA)] for 1h at 37°C. Fluorescence was measured at 485nm (excitation) and 590nm (emission) using SpectraMax Gemini EM spectrophotometer (Molecular Devices, Sunnyvale, CA). The level of ROS is proportional to fluorescence intensity. As a control, 8μl of homogenate from each group was also incubated with the reaction buffer without DCFH-DA. The protein concentration of the supernatant was determined by the Bradford method (Bio-Rad, Hercules, CA), using bovine serum albumin (Sigma-Aldrich, St. Louis, MO) as the standard (Somayajulu et al., 2023). Data were normalized to protein and are represented as mean + SD.

### MitoSOX staining

Mitochondrial ROS was analyzed by fluorescence with a MitoSOX assay (ThermoFisher Scientific) according to the manufacturer’s protocol. Briefly, HCET cells were plated onto coverslips, and treated with 100µg/mL PM_10_ for 6h. Cells were washed with PBS and incubated with 10µM MitoSOX at 37°C for 10 min. Cells were then washed twice with PBS and imaged live using a Zeiss Apotome equipped with an AxioCam HRM camera and processed with photoshop (version 7.0.1). The level of ROS is proportional to fluorescence intensity. All images were acquired at the same magnification and processed similarly.

### GSH assay

Total GSH levels were analyzed by a glutathione assay kit (Cayman Chemical, Ann Arbor, MI) per the manufacturer’s protocol and as described before (Kimura and Kimura, 2004). Briefly, cells (n=3/group/treatment) treated with 100µg/ml PM_10_ ± strain 19660 ± SKQ1 were harvested in 500µl of ice cold 50mM MES (2-(N-morpholino) ethanesulphonic acid) containing 1mM EDTA, homogenized and centrifuged at 10,000g at 4°C for 10min and supernatant collected. Total GSH levels were determined by Ellman’s reagent using a standard curve per manufacturer’s protocol and then normalized to total protein in each sample. Final GSH levels were expressed as mean + SD.

### MDA assay

Lipid peroxidation was examined by measuring the malondialdehyde (MDA) levels by a TBARS assay kit (Cayman Chemical, Ann Arbor, MI) per the manufacturer’s protocol. Briefly, cells (n=3/group/treatment) treated with 100µg/ml PM_10_ ± strain 19660 ± SKQ1 were harvested in 500µl of ice cold PBS with protease inhibitor, homogenized and centrifuged at 10,000g at 4°C for 10mins and supernatant collected. The supernatants were incubated with thiobarbituric acid (TBA) for 1h at 90°C under acidic conditions. The MDA-TBA adduct formed was measured calorimetrically at 53-540nm. MDA levels were calculated using a standard curve per manufacturer’s protocol and then normalized to total protein in each sample. MDA levels were expressed as mean + SD

### ELISA

An ELISA kit was used to measure protein levels of IL-10 (Duoset ELISA kit, R&D systems, Minneapolis, MN). Briefly, HCET cells were treated as mentioned above and cells (n=3/group/treatment) from the control or 100µg/ml PM_10_ ± strain 19660 ± SKQ1 were lysed in PBS containing 0.1% Tween 20 and protease inhibitors. All assays were run per the manufacturers’ protocol and data were expressed as mean + SD.

### Statistical analysis

*In vivo* comparison of clinical scores between two groups at each time was tested by the Mann-Whitney U test. For *in vitro* studies, significance of RT-PCR between control vs. PM_10_ treated groups was determined by a Student’s t-test. To demonstrate significance between 3 or more groups (*in vitro*), a one-way ANOVA followed by the Bonferroni’s multiple comparison test was used for MTT, GSH, MDA assays, ROS measurements, ELISA and western blots. Data were considered significant at p<0.05. All experiments were repeated at least once to ensure reproducibility and data are shown as mean ± SEM (*in vivo*) and mean +SD (*in vitro*).

## Results

### PM_10_ exposure *in vivo* affects ocular response to infection

**Figure 1:**
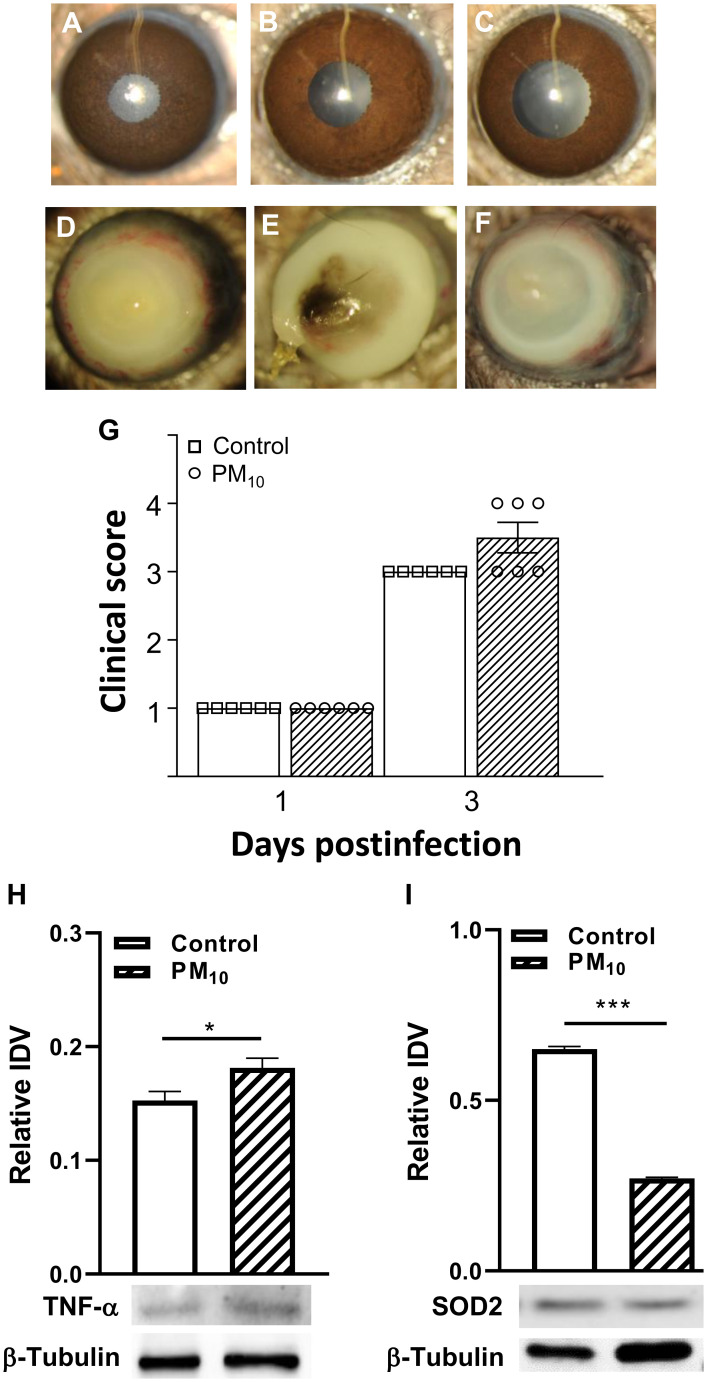
Effects of control air and PM_10_ on mouse corneas infected with strain 19660. **(A-C)** shows normal cornea **(A)**, control air cornea **(B)** and PM_10_ exposed cornea **(C)** are all clear. Slit lamp photographs from control air exposed **(D)** showed opacity over the entire cornea, while PM_10_ exposed showed perforation **(E)**, and central corneal thinning **(F)** at 3 days p.i. (n=5/group/time). Clinical scores **(G)** at 1 and 3 days p.i. No significant differences in clinical scores were observed between control and PM_10_ exposed corneas at 1 day p.i. However, at 3 days p.i., the PM_10_ exposed group had 50% more corneas with a higher clinical score. PM_10_ exposure and infection caused a significant increase in protein levels of pro-inflammatory modulator TNF-α **(H)** and a significant decrease in the anti-oxidant enzyme SOD2 **(I)**. *p<0.05, ***p<0.001.

**Figures 1A–C** show slit lamp photographs of normal cornea (A), cornea exposed to control air (B) and cornea exposed to PM_10_ before infection (C). Corneas (A-C) appear clear. **Figures 1D–F** are slit lamp photographs of typical eyes from *P. aeruginosa* infected mice. PM_10_ exposure (500μg/m^3^) resulted in earlier perforation (**Figure 1E**) or thinning (**Figure 1F**) vs. control air exposure that showed dense opacity (**Figure 1D**) over the entire anterior segment. Clinical scores (**Figure 1G**) showed disease in both control air and PM_10_ exposed infected eyes at 3 days p.i. Although not significant, differences were observed between the two infected groups with a trend of worsening disease in the PM_10_ exposed group (greater than +3 and thinning of the cornea). No differences were observed between infected eyes in control air and PM_10_ exposed groups at day 1 p.i. (**Figure 1G**) where the score was +1 for both groups. **Figures 1H, I** revealed that downstream protein, pro-inflammatory TNF-α is significantly (p<0.05) increased, and the anti-oxidant enzyme, SOD2 is decreased significantly (p<0.001) after PM_10_ exposure and infection vs. control air, setting the stage for the following studies.

### PM_10_ exposure decreases cell viability, anti-oxidant enzyme levels, increases ROS and inflammation *in vitro* in HCET

**Figure 2:**
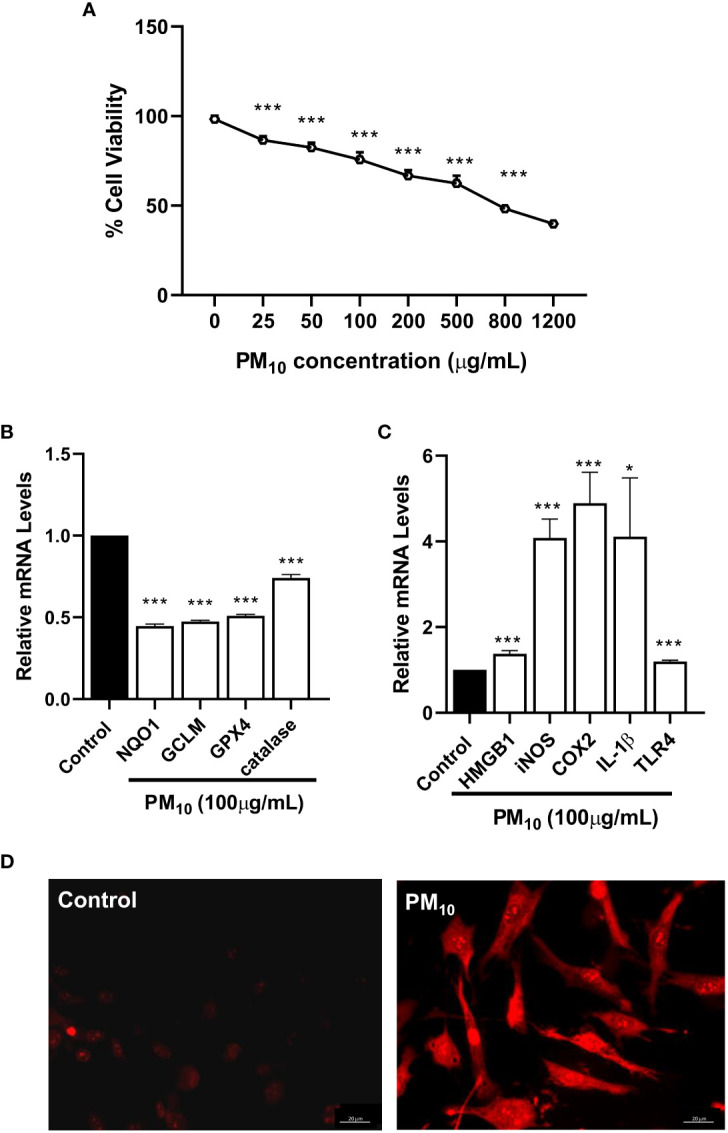
*In vitro* effects of PM_10_ on HCET: cell viability, anti-oxidant enzymes, inflammation and mitochondrial ROS. **(A)**. Cells exposed to PM_10_ for 24h showed a concentration dependent decrease in cell viability measured by MTT assay. At 1200μg/ml, the highest concentration tested, approximately 50% remained viable. **(B)**. RT-PCR showed that PM_10_ exposure (100μg/ml) significantly reduced mRNA levels of antioxidant enzymes compared to control after 24h. **(C)**. RT-PCR showed that PM_10_ exposure (100μg/ml) significantly increased mRNA levels of pro-inflammatory molecules compared to control after 24h. **(D)**. MitoSOX staining shows PM_10_ exposed (100μg/ml) cells have increased fluorescence vs. control indicating higher mitochondrial ROS levels. Scale bar = 20μm. Data are expressed as mean + SD. (*p<0.05, ***p<0.001, n=3).

*In vitro*, the effects of PM_10_ on HCET were evaluated by testing cell viability (**Figure 2A**), ROS levels (**Figure 2B**) and pro-inflammatory modulators (**Figure 2C**). HCET exposed to PM_10_ for 24h showed a concentration dependent decrease (p<0.001) in live cells (**Figure 2A**). At concentrations of 25-100μg/ml, 75-90% of the cells were viable compared to controls. At 1200μg/ml, the highest concentration tested, less than 50% remained viable. **Figure 2B** shows PM_10_ significantly reduced the mRNA levels of anti-oxidant enzymes: NQO1 (p<0.001), GPX4 (p<0.001), catalase (p<0.001) and GCLM (p<0.001). **Figure 2C** indicates increased mRNA levels of pro-inflammatory markers in HCET exposed to PM_10_ for HMGB1 (p<0.001), iNOS (p<0.001), COX2 (p<0.001), IL-1β (p<0.05) and TLR4 (p<0.001 vs. control HCET. ROS production by mitochondria visualized by fluorescence microscopy using MitoSOX red dye is shown in **Figure 2D**. Data clearly show increased fluorescence indicating higher superoxide produced by mitochondria of HCET exposed to PM_10_ compared to control HCET.

### PM_10_ effects cell viability of HCET challenged with *P. aeruginosa*

**Figure 3:**
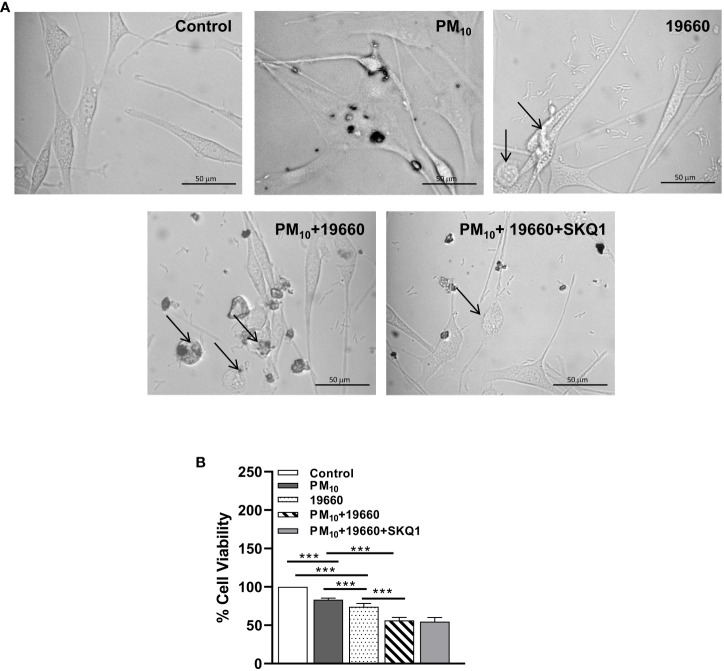
*In vitro* effects of strain 19660 after PM_10_ exposure on HCET: morphology and cell viability. **(A)**. Phase contrast images of cells challenged with strain 19660 after PM_10_ exposure and treatment with SKQ1. Cells in the media control group appear spindle shaped with prominent nuclei. Cells exposed to PM_10_ appear flattened and enlarged. More cells appear rounded and thinned upon 19660 challenge after PM_10_ exposure compared to cells exposed to bacteria alone (arrows). SKQ1 pre-treated cells appear spindle shaped with prominent nuclei and have fewer rounded cells, similar to control. **(B)**. Cells exposed to PM_10_ (100μg/ml) and then challenged with strain 19660 show a significant reduction in cell viability (MTT assay) as compared to control cells. SKQ1 pre-treatment (50nM) doesn’t restore cell viability. Scale bar=50μm. Data are expressed as mean + SD. (***p<0.001, n=3).

The effects of PM_10_ on the response of HCET to *P. aeruginosa* were examined by phase contrast microscopy and cell viability tested. Phase contrast microscopy (**Figure 3A**) clearly shows that controls have prominent nuclei and appear spindle shaped. Cells exposed to PM_10_ appear to be enlarged and flattened with enlarged nuclei. After *P. aeruginosa* challenge, a small number of rounded cells (indicated by arrows) were seen. When PM_10_ exposed cells, were challenged with the bacteria, more cells appeared rounded and nuclei were difficult to observe. Cells pre-treated with SKQ1, a mitochondria targeted antioxidant, appeared spindle-shaped and similar to the control group, with fewer rounded cells. Cell viability data (**Figure 3B**) indicate that compared to controls, exposure to PM_10_ or challenge with strain 19660 significantly lowered HCET viability (p<0.001). Significant additional reduction in cell viability was observed in PM_10_ exposed cells challenged with strain 19660 compared to cells in control, PM_10_ or bacteria challenged groups (p<0.001). SKQ1 pre-treatment did not protect against the PM_10_ induced additional loss in cell viability after bacterial challenge. Similar effects of PM_10_ and SKQ1 were observed on cells challenged with strain 19660 and cell viability was analyzed with the trypan blue dye exclusion test shown in **Supplementary Figure 1**.

### PM_10_ upregulates *P. aeruginosa* induced oxidative stress

**Figure 4:**
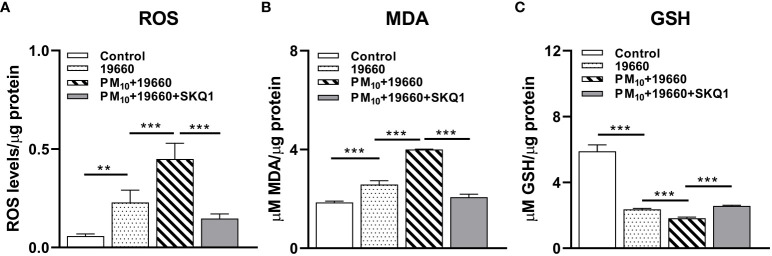
HCET challenged with ATCC 19660 after PM_10_ exposure show exacerbated oxidative stress: ROS, MDA and GSH levels. **(A)**. Elevated ROS level in cells challenged with ATCC 19660 after PM_10_ exposure was significantly reduced by SKQ1. **(B)**. MDA levels were significantly reduced by SKQ1 in cells challenged with ATCC 19660 after exposure to PM_10_. **(C)**. Lowered GSH levels in cells challenged with ATCC 19660 after PM_10_ exposure were significantly rescued by SKQ1. Data are expressed as mean + SD. (**p<0.01, ***p<0.001, n=3).

We have previously shown that PM_10_ exposure induces oxidative stress in HCET (Somayajulu et al., 2023). To assess the effects of PM_10_ on *P. aeruginosa* induced oxidative stress the levels of ROS (**Figure 4A**), lipid peroxidation end product MDA (**Figure 4B**) and GSH (**Figure 4C**) were analyzed. Data show that compared to control, ROS levels (**Figure 4A**) were significantly elevated (p<0.01) in strain 19660 challenged cells. PM_10_ exposure significantly upregulated this increase (p<0.001) in ROS levels in cells challenged with bacteria. Pre-treatment with SKQ1 significantly reduced ROS levels (p<0.001) in bacteria challenged cells exposed to PM_10_. **Figure 4B** shows significantly elevated levels of MDA (p<0.001 after cells were challenged with strain 19660 vs. control. Exposure to PM_10_ before bacterial challenge caused an additional increase in MDA levels compared to bacterial challenge alone (p<0.001). SKQ1 pretreatment reduced the MDA levels significantly (p<0.001) in PM_10_ exposed cells challenged with strain 19660. The effects of SKQ1 on MDA levels in cells exposed to only PM_10_ or bacteria alone are shown in **Supplementary Figure 2A**. **Figure 4C** demonstrates that GSH levels were significantly reduced in cells challenged with bacteria vs. control (p<0.001). PM_10_ exposure prior to bacterial challenge caused an additional reduction in GSH levels (p<0.001) vs. bacteria alone. SKQ1 pre-treatment was able to significantly elevate (p<0.001). PM_10_ mediated additional loss in GSH levels. The effects of SKQ1 on GSH levels in cells exposed to only PM_10_ or bacteria alone are shown in **Supplementary Figure 2A**.

#### PM_10_ affects the levels of anti-oxidant enzymes in cells challenged with *P. aeruginosa*

**Figure 5:**
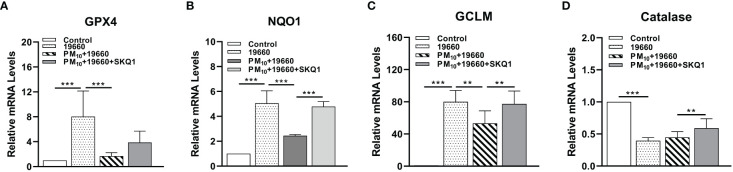
Effects of strain19660, PM_10_ and SKQ1 on mRNA levels of antioxidant enzymes. RT-PCR showed significantly lowered mRNA levels for GPX4 **(A)**, NQO1 **(B)**, GCLM **(C)**, and catalase **(D)** in cells challenged with strain 19660 after PM_10_ exposure compared to cells challenged with bacteria alone. These levels were significantly restored by SKQ1. Data are expressed as mean + SD. (**p<0.01, ***p<0.001, n=3).

mRNA levels of anti-oxidant enzymes measured by RT-PCR are represented in **Figure 5**. Cells challenged with bacteria vs. control showed a significant increase in the levels of GPX4 (A, p<0.001), NQO1 (B, p<0.001), GCLM (C, p<0.001). However, mRNA levels of catalase were significantly lower in bacteria challenged cells vs. controls (D, p<0.001). When *P. aeruginosa* challenge was accompanied by PM_10_ exposure, a significant downregulation was observed in the expression levels of GPX4 (A, p<0.001), NQO1 (B, p<0.001), GCLM (C, p<0.01) compared to bacteria alone. PM_10_ exposure did not affect catalase mRNA levels in strain19660 challenged cells vs. those challenged with bacteria alone. Cells treated with SKQ1 prior to PM_10_ exposure and bacterial challenge had significantly higher levels of only NQO1 (B, p<0.001), GCLM (C, p<0.01) and catalase (D, p<0.01).

#### PM_10_ induces increased cell inflammation responses in cells challenged with *P. aeruginosa*

**Figure 6:**
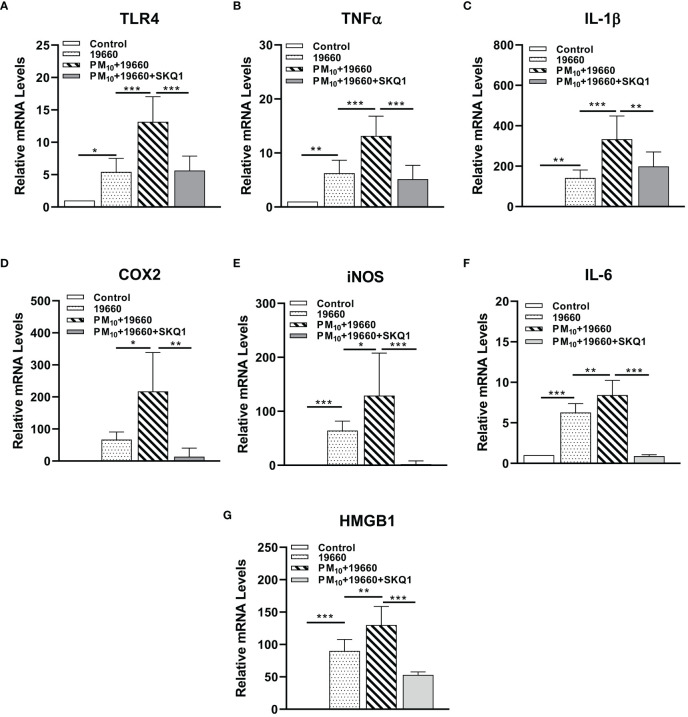
mRNA levels of pro-inflammatory markers are increased in cells challenged with strain 19660 after PM_10_ exposure: protection by SKQ1. RT-PCR showed significantly elevated mRNA levels for TLR4 **(A)**, TNF-α **(B)**, IL-1β **(C)**, COX2 **(D)**, iNOS **(E)**, IL-6 **(F)** and HMGB1 **(G)** in cells challenged with ATCC 19660 after PM_10_ exposure compared to cells challenged with bacteria alone. SKQ1 pre-treatment reversed these effects. Data are expressed as mean + SD. (*p<0.05, **p<0.01, ***p<0.001, n=3).

**Figure 7:**
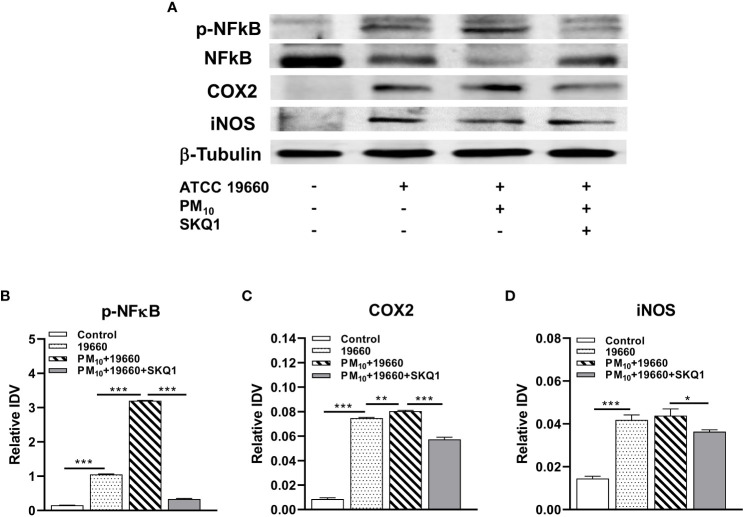
Protein levels of pro-inflammatory markers are increased in cells challenged with strain 19660 after PM_10_ exposure: protection by SKQ1. **(A)**. Western blots used to determine the levels of pNFκB, NFκB, COX2 and iNOS. Densitometry showed significantly elevated protein levels for pNFκB **(B)**, COX2 **(C)** and iNOS **(D)** in cells challenged with strain 19660 after PM_10_ exposure, which were significantly lowered by SKQ1. Data are expressed as mean + SD. (*p<0.05, **p<0.01, ***p<0.001, n=3).

mRNA levels of pro-inflammatory markers measured by RT-PCR are represented in **Figure 6** and protein levels analyzed by western blot are shown in **Figures 7A-D**. Cells challenged with bacteria vs. control demonstrated a significant increase in the level of only TLR4 (A, p<0.05), TNF-α (B, p<0.01), IL-1β (C, p<0.01), iNOS (E, p<0.001), IL-6 (F, p<0.001) and HMGB1 (G, p<0.001). A significantly upregulation in the expression level of TLR4 (A, p<0.001), TNF-α (B, p<0.001), IL-1β (C, p<0.001), COX2 (D, p<0.05), iNOS (E, p<0.05), IL-6 (F, p<0.01) and HMGB1 (G, p<0.01) was observed in the *P. aeruginosa* challenged group that was exposed to PM_10_, compared to the group challenged with bacteria alone. Cells treated with SKQ1 before PM_10_ exposure and bacterial challenge had significantly lower levels of TLR4 (A, p<0.001), TNF-α (B, p<0.001), IL-1β (C, p<0.01), COX2 (D, p<0.01), iNOS (E, p<0.001), IL-6 (F, p<0.001) and HMGB1 (G, p<0.001) compared vs. those without SKQ1. **Figure 7A** shows western blot analysis of PM_10_ exposed cells challenged with bacteria in the presence and absence of SKQ1. Relative IDV values (7B-D) indicate significantly elevated protein levels of p-NFκB (B, p<0.001), COX2 (C, p<0.001) and iNOS (D, p<0.001) in cells challenged with bacteria vs. control. PM_10_ exposure of bacteria challenged cells caused a further upregulation in the levels of p-NFκB (B, p<0.001), COX2 (C, p<0.01) but not iNOS. Cells pre-treated with SKQ1 before PM_10_ and bacteria showed a significant reduction in the levels of p-NFκB (A, p<0.001), COX2 (C, p<0.001) and iNOS (D, p<0.05) compared to those without SKQ1.

#### IL-10 levels are lowered by PM_10_

**Figure 8:**
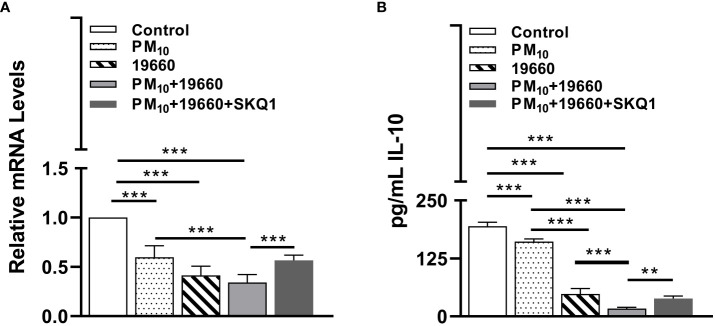
IL-10 levels are decreased in cells challenged with strain 19660 after PM_10_ exposure and SKQ1 reverses this effect. RT-PCR showed mRNA **(A)** and ELISA showed protein **(B)** levels of IL-10 were significantly lowered in cells challenged with strain 19660 after PM_10_ exposure compared to cells challenged with bacteria alone. SKQ1 reversed the loss of IL-10 at both mRNA and protein level. Data are expressed as mean + SD. (**p<0.01, ***p<0.001, n=3).

IL-10 is an anti-inflammatory cytokine which plays an important role in infection by regulating the immune response against pathogens and thus avoiding damage to the host (Saraiva and O'Garra, 2010). The mRNA and protein levels of IL-10 in cells exposed to PM_10_ and challenged with bacteria are represented in **Figures 8A, B**. **Figure 8A** shows that compared to controls, cells exposed to PM_10_ or challenged with bacteria alone and bacteria plus PM_10_ had significantly reduced (p<0.001) IL-10 mRNA levels. A slight but not significant decline in IL-10 mRNA levels was observed in cells challenged with bacteria after PM_10_ exposure vs. cells challenged with bacteria alone. SKQ1 pre-treatment before PM_10_ and bacteria showed a significant (p<0.001) restoration of IL-10 mRNA compared to without SKQ1. **Figure 8B** demonstrates the levels of IL-10 protein in different treatment groups. Cells exposed to only PM_10_ or challenged with 19660 alone, showed a significant reduction (p<0.001) in IL-10 protein compared to control. Cells challenged with 19660 after PM_10_ exposure also displayed a significant decline (p<0.001) in IL-10 protein compared to control cells, PM_10_ exposed cells, and cells challenged with 19660 alone. SKQ1 pre-treatment significantly reversed (p<0.01) the decline in IL-10 protein in cells exposed to PM_10_ before strain 19660 challenge.

## Discussion

The world’s population, increasingly living in urban areas, is anticipated to reach 68% by 2050 (Sendra et al., 2021) and exposure to air pollution in the urban setting is a major risk factor for disease. One of the major components of air pollution is particulate matter (PM), which is a mixture of liquid and solid particles of various sizes and chemical properties (Li et al., 2022). The negative impact of particulate matter on the human body is mainly from PM_2.5_ and PM_10_ (Li et al., 2022), with adverse events linked to cardiovascular and pulmonic diseases. Exposure to PM is also linked to dry eye (Li et al., 2017), ocular discomfort, (Novaes et al., 2010), abnormalities in tear film, reduced tear break-up time and other subclinical alterations of the ocular surface (Novaes et al., 2007). In particular, as part of the ocular surface, the cornea which must remain optically clear for good vision, is constantly exposed to the environment and serves as a barrier against the entry of harmful pathogens (Zhou et al., 2022). Exposure to particulate has been shown to compromise the barrier function in a reconstructed human corneal epithelial model by reducing the levels of proteins such as occluding zonules that are a component of the cells tight junction complex, required for maintaining tissue integrity (Alfuraih et al., 2020). Additionally, PM induced compromise in barrier function has been shown to promote *P. aeruginosa* infection in other tissues exposed to particulates, such as the lung (Liu et al., 2019). Whether or not exposure of the eye to particulates has a detrimental impact on the development/progression of ocular infectious diseases such as *P. aeruginosa* induced bacterial keratitis are currently unknown and is the purpose of this study.

In the current study, we analyzed the impact of acute exposure to PM_10_ on *P. aeruginosa* induced keratitis and also studied the ameliorative effects of SKQ1, a mitochondrial anti-oxidant. We used a whole-body exposure chamber to subject mice to a dose of PM_10_ of 500μg/m^3^ before bacterial challenge. Normally, in this model, mouse corneas perforate by day five (Hazlett et al., 2001), however, in this study we observed that they were thinned and/or perforated by day 3 after PM_10_ exposure. This was accompanied by significantly elevated levels of TNF-α and reduced levels of SOD2 protein that are indicative of inflammation and oxidative stress. These findings are consistent with a mouse study of herpes simplex virus (HSV)-1 induced keratitis, where mouse corneas exposed to polluted air from the city of Buenos Aires showed a more severe disease outcome than controls, including increased corneal opacity and pro-inflammatory cytokine production (Sendra et al., 2021).

This study extends previous work in which we examined the effects of topical application of PM_2.5_ (400μg/ml) post infection with *P. aeruginosa* on the mouse cornea (Somayajulu et al., 2020). Application of PM_2.5_ 6h post infection resulted in severe corneal disease, including early perforation and worsened clinical scores at 2 days post infection (Somayajulu et al., 2020). However, the PM_2.5_ model had drawbacks including that it was a drop method vs. whole-body exposure and we could find no ties to oxidative stress as a contributory factor. Therefore, the model did little to clarify any mechanisms underlying the effects of particulates on keratitis and how they possibly were connected to oxidative stress. Since the main hypothesis of this study is that PM_10_ induces oxidative stress, which leads to inflammation that makes the cornea more susceptible to infection this was a major drawback. To uncover the mechanism underlying early perforation in the mouse cornea by PM_10_, we turned to an *in vitro* model which can be better controlled, where HCET were exposed to PM_10_ prior to bacterial challenge with strain 19660.

The toxic effects of PM_10_ include: cell viability loss, increased oxidative stress, inflammation and impaired barrier function and have been well established in different *in vitro* models (Li et al., 2008; Chirino et al., 2010; Yoon et al., 2018; Ko et al., 2020; Aghapour et al., 2022). Our analysis showed that PM_10_ caused a dose dependent loss in cell viability, reduced anti-oxidant enzyme levels, and increased ROS and pro-inflammatory modulators. Our data are consistent with a previous study which demonstrated that PM_10_ isolated from road dust in Seoul, South Korea decreased cell viability and increased ROS production and inflammation in HCET (Yoon et al., 2018). Other studies have also confirmed these toxic effects of PM_10_ in human lung epithelial cells (Chirino et al., 2010) and alveolar epithelial cells (Ko et al., 2020). Oxidative stress is caused by an imbalance between ROS production and anti-oxidant defenses (Pizzino et al., 2017). The detrimental effects of PM_10_ on the anti-oxidant defense system (Hatzis et al., 2006) have also been well established (Chirino et al., 2010). Studies have shown that PM_10_ lowered GSH levels in lung epithelial cells, which resulted in increased lipid peroxidation and protein oxidation (Chirino et al., 2010). Additionally, more recently, we have shown that PM_10_ reduces Nrf2 levels in both primary and immortalized human corneal epithelial cells (Somayajulu et al., 2023). Nrf2 is a master regulator of the anti-oxidant defense mechanism and is activated upon oxidative stress, in turn activating many genes, including NQO1, GCLM, and GPX4 (Pardo et al., 2020) that encode for anti-oxidant enzymes. When we measured the levels of these enzymes, we observed that PM_10_ reduced their levels in HCET, further strengthening our hypothesis that PM_10_ induces oxidative stress in these cells. PM-induced oxidative stress also increases inflammatory modulators such as TNF-α, IL-1β, IL-6, IL-8, and COX2 (Wang et al., 2017; Yoon et al., 2018; Radan et al., 2019; Ko et al., 2020; Pintha et al., 2021). When we measured this class of modulators, we observed a PM_10_ mediated upregulation of IL-1β, iNOS, COX2, HMGB1 and TLR4 in HCET. Our data suggest that PM_10_ exposure causes a pro-oxidant and pro-inflammatory milieu in the cells. To further test that PM_10_ exacerbates the effects of bacterial infection; we challenged cells with *P. aeruginosa* after PM_10_ exposure and also tested the effects of the anti-oxidant SKQ1 in HCET.

We observed that PM_10_ exposure before bacterial challenge served to further elevate *P. aeruginosa* induced loss in cell viability, and oxidative stress (increased ROS production, lipid peroxidation and lowered GSH levels). Furthermore, we found that the anti-oxidant SKQ1 reversed these effects on oxidative stress by reducing ROS levels and lipid peroxidation while restoring GSH levels. These findings are consistent with former reports demonstrating that urban particulate matter further escalates *P. aeruginosa* (strain PAO1) induced oxidative stress and ROS production (Chen et al., 2018) in human bronchial epithelial cells, which was reversed by N-acetylcysteine (Chen et al., 2018), which has anti-inflammatory and anti-oxidant capabilities. However, *P. aeruginosa* produces many toxic compounds, such as pyocyanin that can directly oxidize GSH and reduce its levels, producing ROS in airway epithelial cells (O'Malley et al., 2004). These findings suggest that cells infected with bacteria are already under oxidative stress and the presence of PM_10_ further escalates it. In response to oxidative stress, the cells deploy anti-oxidant defense mechanism based on enzymatic components which serve to protect them from ROS mediated damage (Deponte, 2013). In this study, the measurement of anti-oxidant enzyme levels showed increased GPX4, NQO1 and GCLM levels after bacterial challenge, which is indicative of the normal response to oxidative stress. However, cells exposed to PM_10_ before bacterial challenge showed a marked reduction of these anti-oxidant enzymes, suggesting that PM_10_ impaired the anti-oxidant response causing additional oxidative stress in these cells.

One of the responses to PM-mediated oxidative stress is inflammation (Wang et al., 2017). When we analyzed different immune modulators and cytokines in response to bacterial challenge after PM_10_ exposure, we saw an upregulation of pro-inflammatory cytokines IL-6, IL-1β, and TNF-α and other modulators: NF-κB, COX-2, iNOS and TLR4. Our data are similar to previous studies in alveolar macrophages stimulated with PM_2.5_ and challenged with *P. aeruginosa* (PAO1) that showed an upregulation of similar pro-inflammatory cytokines such TNF-α, IL-6, and NF-κB pathway-related proteins (Liu et al., 2019). We also observed that while PM_10_ increased the levels of pro-inflammatory cytokines, it reduced the levels of the anti-inflammatory cytokine IL-10 in cells challenged with bacteria. These findings are consistent with former reports in human bronchial epithelial cells exposed to urban particulate matter before infection with bacteria that caused an upregulation of pro-inflammatory cytokine IL-8, while downregulating anti-inflammatory cytokine IL-13 (Chen et al., 2018). Our hypothesis that oxidative stress induced by PM_10_ mediates inflammation is further supported by the fact that SKQ1 abrogated the increased levels of TNF-α, IL-6 and IL-1β, and restored IL-10 levels in cells challenged with bacteria and PM_10_. The ameliorative effects of SKQ1, a mitochondria targeted anti-oxidant against PM_10_ mediated toxicity, have been previously demonstrated by us in both primary and immortalized human corneal epithelial cells (Somayajulu et al., 2023).

We have used PM_10_ by direct application into the culture medium where we have established a functional model. However, the generalizability of the findings may prove different compared to real world scenarios in which the whole body is exposed to the particulate. The data implies that exposure to PM_10_ renders the eye more susceptible to infection and thus the caution is for the public to be aware that not only respiratory disease is possible with exposure. that the eye is made more vulnerable to pathogens as well. In fact exposure below 50 μg/m3 (Mu et al., 2022) is a recommended to reduce health risks whereas amounts of 494-1200 μg/m3 are observed in China and India. Thus, the mechanism of PM_10_ activity enhancing *P. aeruginosa* keratitis have been shown in a well-controlled *in vitro* model, but the data need to be verified by *in vivo* studies which are underway, to provide further understanding of the adverse effects of PM_10_ on keratitis.

In conclusion, PM_10_ impairs the anti-oxidant defense mechanism leading to increased oxidative stress, aggravating inflammation and triggering increased susceptibility to infection. The adverse effects of oxidative stress are reversed by using the anti-oxidant SKQ1 which targets mitochondrial ROS. These findings provide insight into the underlying mechanisms of early perforation and increased disease severity observed in *P. aeruginosa* infected corneas exposed to PM_10_.

## Data availability statement

The raw data supporting the conclusions of this article will be made available by the authors, without undue reservation.

## Ethics statement

The animal study was approved by IACUC review board Wayne State University School of Medicine. The study was conducted in accordance with the local legislation and institutional requirements.

## Author contributions

Conceptualization: LH; methodology: MS, SM, and LH; formal analysis: MS, RW, FM, and SM; investigation: MS, SM, RW, and FM; resources: LH; data curation: MS; writing—original draft preparation: MS and LH; writing—review and editing: MS, SM, and LH; visualization: MS and SM; supervision: LH; project administration: LH; funding acquisition: LH. All authors contributed to the article and approved the submitted version.
