# Supplementary material for: PM10 and Pseudomonas aeruginosa: effects on corneal epithelium
Source: Front Cell Infect Microbiol. 2023 Oct 5;13:1240903. doi: 10.3389/fcimb.2023.1240903 (PMC10585254; doi:10.3389/fcimb.2023.1240903)
Supplement: Supplementary file 1 [file Image_1.pdf]

Supplementary Material

PM_{10} and *Pseudomonas aeruginosa*: Effects on corneal epithelium

Mallika Somayajulu[1], Sharon A McClellan[1], Farooq Muhammed[1], Robert Wright[1], and Linda D Hazlett[1*]

*** Correspondence:** Corresponding Author: lhazlett@med.wayne.edu

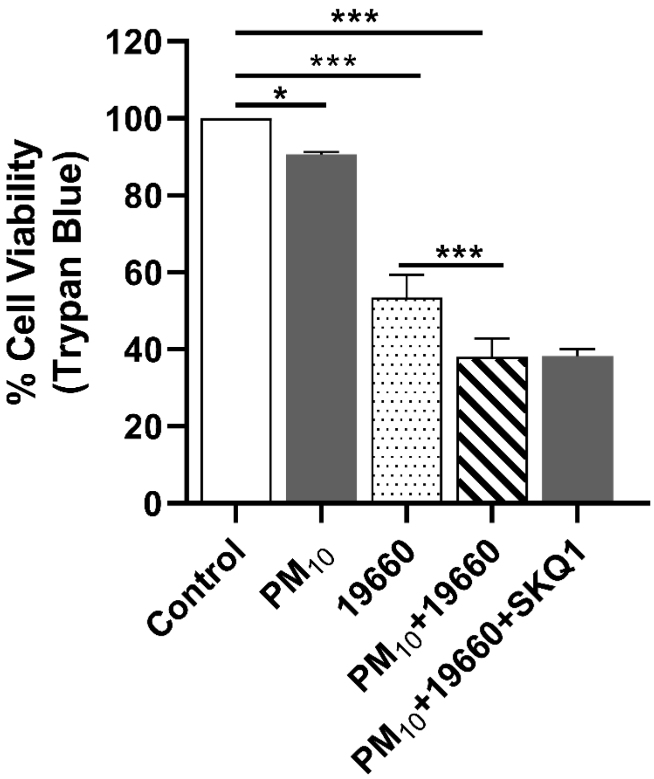

Supplementary Figure 1. *In vitro* effects on cell viability after challenge with strain 19660 and PM_{10} exposure on HCET. Trypan blue exclusion test showed that cells exposed to PM_{10} (100µg/ml) and then challenged with strain 19660 show a significant reduction in cell viability compared to control cells. SKQ1 pre-treatment (50nM) fails to restore cell viability. (*p<0.05, ***p<0.001, n=3)

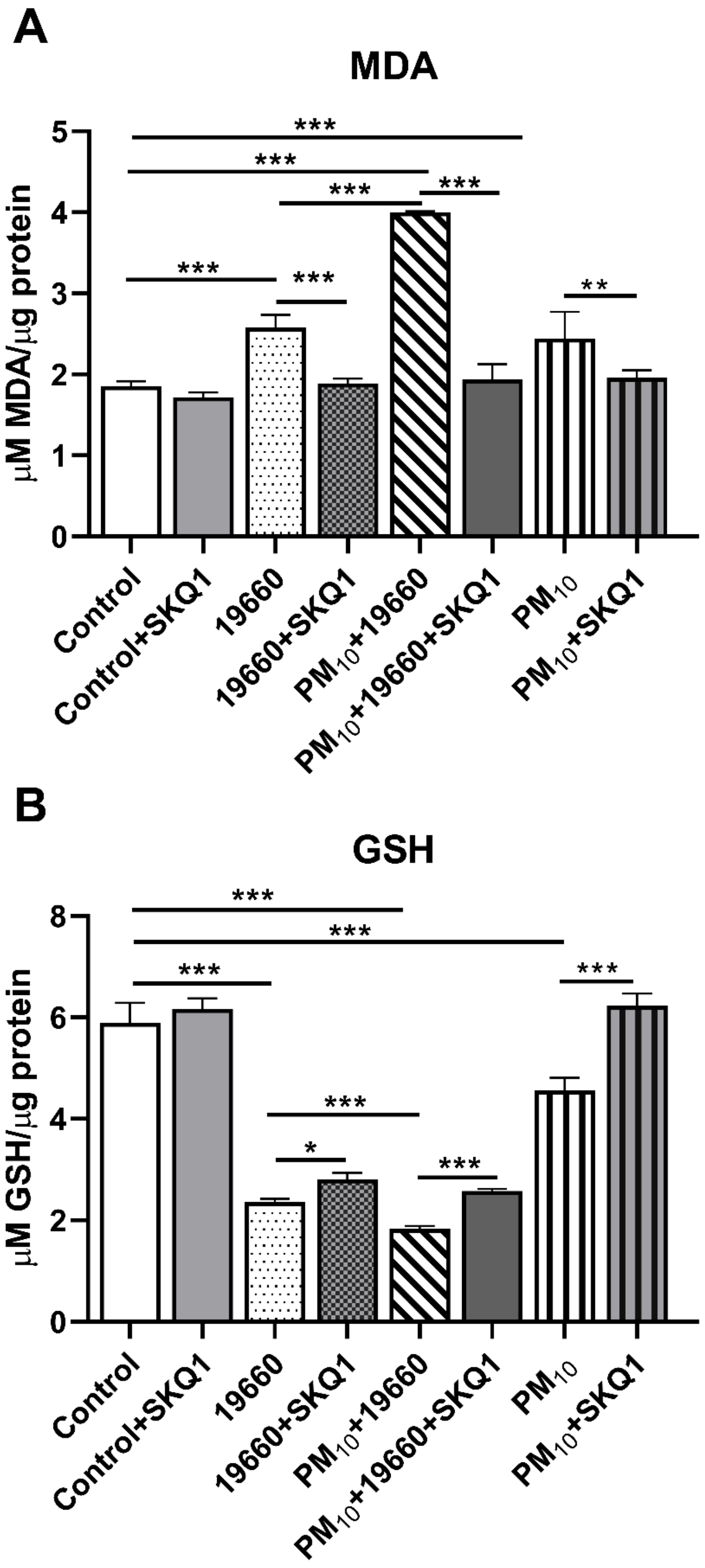
A
MDA
µM MDA/µg protein
0
1
2
3
4
5
***
***
***
***
***
***
**
Control
Control+SKQ1
19660
19660+SKQ1
PM10+19660
PM10+19660+SKQ1
PM10
PM10+SKQ1
B
GSH
µM GSH/µg protein
0
2
4
6
8
***
***
***
***
*
***
***
Control
Control+SKQ1
19660
19660+SKQ1
PM10+19660
PM10+19660+SKQ1
PM10
PM10+SKQ1

Supplementary Figure 2. HCET challenged with ATCC 19660 after PM_{10} exposure show exacerbated oxidative stress and reduced anti-oxidant levels. (A). MDA levels were significantly reduced by SKQ1 in cells exposed to only PM_{10}, challenged with bacteria alone, or challenged with 19660 after exposure to PM_{10}. (B). Lowered GSH levels in cells exposed to only PM_{10}, challenged with bacteria alone or challenged with 19660 after PM_{10} exposure were significantly increased by SKQ1. Data are expressed as mean + SD. (*$p<0.05$, **$p<0.01$, ***$p<0.001$, n=3)
